# Adaptive genome evolution and metabolic rewiring in the highland poppy *Meconopsis integrifolia*

**DOI:** 10.1093/hr/uhag045

**Published:** 2026-03-02

**Authors:** Lamu YangJin, Yi Yuan, Siheng Zeng, Guojin Hu, Mingyang Du, Huiyan Xiong, Xianqing Jia, Ruijun Duan

**Affiliations:** College of Eco-Environmental Engineering, Qinghai University, Xining 810000, China; Key Laboratory of Resource Biology and Biotechnology in Western China, Ministry of Education, Provincial Key Laboratory of Biotechnology of Shanxi Province, College of Life Sciences, Northwest University, Xi’an 710069, China; College of Eco-Environmental Engineering, Qinghai University, Xining 810000, China; College of Eco-Environmental Engineering, Qinghai University, Xining 810000, China; College of Eco-Environmental Engineering, Qinghai University, Xining 810000, China; College of Agriculture and Animal Husbandry, Qinghai University, Xining 810000, China; Key Laboratory of Resource Biology and Biotechnology in Western China, Ministry of Education, Provincial Key Laboratory of Biotechnology of Shanxi Province, College of Life Sciences, Northwest University, Xi’an 710069, China; College of Eco-Environmental Engineering, Qinghai University, Xining 810000, China; College of Agriculture and Animal Husbandry, Qinghai University, Xining 810000, China

Dear Editor,

*Meconopsis* is the second-largest genus in the Papaveraceae family and is widely recognized as the ‘Himalayan poppy’ for its striking, brilliantly colored flowers and high ornamental value. Beyond its horticultural importance, *Meconopsis* is a key component of the alpine flora of the Pan-Himalayan region, with nearly all species endemic to the Qinghai-Tibet Plateau (QTP). The QTP, known as the ‘Roof of the World’, hosts one of Earth’s most extreme ecosystems, characterized by chronic cold, intense ultraviolet radiation, hypoxia, and highly unpredictable pollination opportunities. Plants that successfully colonize these environments must evolve complex structural, physiological, and metabolic strategies [1], and *Meconopsis* species represent one of the most iconic examples of such alpine adaptation [2]. As a traditional Tibetan medicinal genus enriched in flavonoids, alkaloids, and other bioactive compounds, *Meconopsis* also possesses significant pharmacological potential [2]. However, despite its ecological and medicinal importance, genomic resources for this genus have remained absent.

Here, we present the first chromosome-scale genome assembly of *Meconopsis integrifolia*, one of the most widely distributed and morphologically distinctive species in the genus, known as the ‘Highland Beauty’ and characterized by large yellow flowers, dense trichomes, and an unusual foul floral odor that contrasts sharply with the typically fragrant blooms of most angiosperms (Fig. 1A). Using a combination of Illumina short reads, PacBio HiFi long reads, and Hi-C chromatin interaction data, we generated a high-quality 7.28 Gb chromosome-level assembly with high contiguity (scaffold N50 = 172.3 Mb) and completeness (97.0% BUSCO recovery) (Fig. 1B). We annotated 83 495 protein-coding genes and over 14 000 non-coding RNAs, based on integrated homology-based, *ab initio*, and transcriptomic evidence (Fig. 1C). Repetitive sequences account for 90.97% of the *M. integrifolia* genome and are dominated by long terminal repeat retrotransposons (LTR-RTs), whose extensive accumulation has driven pronounced genome expansion, resulting in a substantially larger genome and higher repeat content than those of other Papaveraceae species and previously reported alpine plants (Fig. 1C and 1D). Consistent with recent LTR activity, *M. integrifolia* exhibits a relatively low ratio of solo to intact LTRs (1.25) (Fig. 1E).

**Figure 1:**
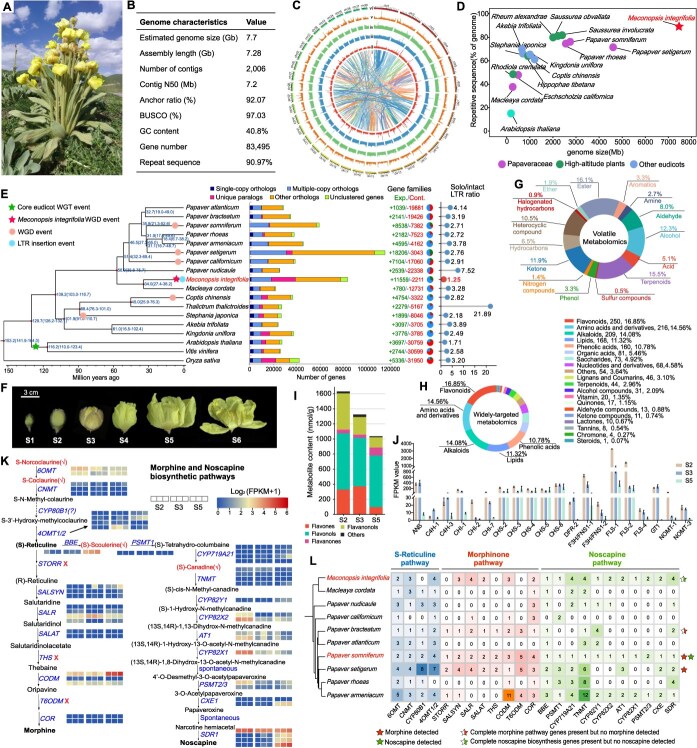
Assessment of *M. integrifolia* genome assembly, annotation, analysis of petal metabolites, and evolution of morphine and noscapine biosynthesis. (A) Phenotypic traits of *M. integrifolia*, exhibiting its rosette growth habit, erect stem, and terminal flowers, with the entire plant covered in golden-yellow trichomes. (B) Assembly and annotation statistics of *M. integrifolia* genome. (C) *De novo* genome assembly and sequencing map of *M. integrifolia*. (i, chromosome size; ii-v, density distributions of genes, GC content, LTR retrotransposons, and tandem repeats (TRF); vi, homologous syntenic blocks among *M. integrifolia* chromosomes). (D) Bubble plot illustrating genome size and repetitive sequence proportions in Papaveraceae, other high-altitude plants, and other eudicot species. (E) Integrated phylogenetic framework with gene family and LTR dynamics. Left: phylogenetic relationships and divergence times (Mya) of *M. integrifolia* and representative species; middle: number of orthologous gene clusters and expansion and contraction of gene families in *M. integrifolia* and other plants, green, expansion; red, contraction; right: ratio of solo to intact LTRs. (F) Flower developmental stages S1-S6 (S1: flower bud stage, S2: flower fully closed stage, S3: flower beginning to crack stage, S4: early flowering stage, S5: flower opening stage, and S6: flower senescing stage). (G) Classification of volatile compounds detected in petals. (H) Classification of widely-targeted metabolites detected in S5 stage petals. (I) Categorization of flavonoid compounds from targeted metabolomics in S2, S3, and S5 stage petals. (J) Expression profiles of flavonoid-related genes across three petal developmental stages. (K) Proposed biosynthetic pathways for morphine and noscapine. Metabolic compounds marked with red checkmarks were detected in *M. integrifolia* petals. The heatmap of Log_2_(FPKM+1) values for morphine and noscapine biosynthetic genes in *M. integrifolia* petals at stages S2, S3, and S5. (L) Copy number variation of morphine and noscapine biosynthesis-related genes in *M. integrifolia* and other Papaveraceae species.

Comparative genomic analyses revealed extensive genome restructuring relative to *Papaver* species, suggesting lineage-specific chromosomal reshuffling following the divergence of *Meconopsis* from other Papaveraceae approximately 34 Mya (Fig. 1E). Synonymous substitution rate (Ks) analyses further identified a *Meconopsis*-specific whole-genome duplication (WGD) event occurring ~6.2 Mya, later than the WGD in *P. somniferum* but earlier than that in *P. setigerum* (Fig. 1E). In addition, analyses of orthologous genes and gene families revealed an increased number of unique paralogs, accompanied by more frequent gene family expansions and fewer contractions (Fig. 1E), suggesting an enhanced potential for functional innovation and adaptive diversification in *M. integrifolia*.

One of the most striking traits of *M. integrifolia* is its unusual floral odor, which is locally perceived as unpleasant and foul and has earned the species the nickname ‘stinking peony’, a feature atypical of showy alpine flowers. To clarify the chemical and molecular basis of this scent, we conducted volatile metabolomic profiling across six developmental stages from early bud to senescence (Fig. 1F). In total, 787 volatile organic compounds (VOCs) were identified. While esters, terpenoids, alcohols, and heterocyclic compounds represented the most abundant groups, odor contribution was dominated by a subset of high-impact volatiles identified using relative odor activity values (rOAVs) (Fig. 1G). More than 390 VOCs showed significant contributions to the scent profile, including ethers, ketones, and nitrogen-containing compounds typically associated with decay-like or animal-associated odors (Fig. 1G). These patterns, together with field observations of insect visitation under high-altitude conditions with limited pollinator diversity, suggest that malodorous volatiles may serve as an adaptive strategy to attract specialized pollinators (e.g., carrion-associated flies) in environments where sweet-scent-attracted pollinators are scarce [3].

In addition to floral scent, we examined the biochemical and transcriptional mechanisms underlying yellow petal pigmentation. A widely targeted metabolomic analysis of petals at the S5 stage revealed high accumulation of flavonoids, amino acids and derivatives, alkaloids, lipids, and phenolic acids (Fig. 1H). Further flavonoid-targeted metabolic profile showed that the content of flavonoids gradually decreased during petal development, with flavonols being the most abundant, followed by flavanonols, flavones, and flavanones, while other flavonoid compounds contributed minimally (Fig. 1I). Transcriptomic profiling identified strong expression of *CHS*, *CHI*, *F3H*, and downstream genes associated with the chalcone-flavonol branch, along with carotenoid biosynthetic components such as *PSY* and *LCYB* (Fig. 1J). However, several anthocyanin pathway genes, particularly *DFR* and *ANS*, showed minimal expression, likely contributing to the absence of red or blue pigmentation (Fig. 1J). Combined metabolomic-transcriptomic evidence suggests that petal coloration is shaped by the interplay of a specialized flavonoid-chalcone pathway and a partially active carotenoid network. The predominance of yellow pigmentation may reflect ecological selection for enhanced UV protection, thermal regulation, or pollinator attraction under alpine conditions [3].

Given the prominent medicinal value of benzylisoquinoline alkaloids (BIAs), particularly the morphinan subclass that includes the clinically important compounds morphine and noscapine (Fig. 1K), we sought to elucidate why *Meconopsis* species lack the capacity for morphinan and noscapine biosynthesis despite their close phylogenetic relationship to *Papaver*. Genome-wide analyses revealed that genes involved in the early steps of the BIA pathway, including *TYDC*, *NCS*, and *6OMT*, are conserved in *Meconopsis* and exhibit detectable transcriptional activity (Fig. 1K and 1L), indicating that the upstream BIA metabolic framework remains largely intact. In striking contrast, several key enzymes (STORR, THS, T6ODM) that are strictly required for morphine and noscapine biosynthesis were absent or nonfunctional (Fig. 1L). Notably, STORR, a lineage-specific fused enzyme in *Papaver* that catalyzes the stereochemical conversion of (S)-reticuline to (R)-reticuline and serves as an essential metabolic gatekeeper for morphinan production [4, 5], was completely missing from the *Meconopsis* genome (Fig. 1L). Consistent with this loss, multiple downstream genes responsible for the biosynthesis of thebaine, codeine, and morphine were either absent or pseudogenized. Transcriptomic evidence further supports this conclusion, as the majority of these downstream morphinan-related or noscapine-related genes exhibited extremely low or undetectable expression levels (Fig. 1K). These restrictions likely represent an evolutionary trajectory in which *M. integrifolia* has not evolved the capacity to synthesize morphine and noscapine, despite retaining upstream precursor production. This metabolic rewiring provides a genomic explanation for the absence of morphinan alkaloids in *Meconopsis* and highlights the evolutionary plasticity of specialized metabolism within the Papaveraceae.

Together, this study provides the first chromosome-scale genomic framework for *M. integrifolia*, and integrates multi-omics analyses to investigate the molecular basis of its key adaptive distinctive traits, including its unique malodorous volatiles, its yellow petal pigmentation, and its inability to synthesize morphine and noscapine despite being closely related to *Papaver* species that produce these medically valuable BIAs. These resources provide critical insights into genome gigantism, metabolic rewiring, and floral adaptation in *M. integrifolia*, and establishes a valuable genomic resource for understanding the evolution of alpine plant evolution.

## Data Availability

The genome raw sequences reads and raw RNA-seq data described in this article were submitted to NCBI with the BioProject number PRJNA1263796 and the BioSample number SAMN48537741. Genome assembly and annotations were also submitted to NGDC Genome Warehouse database (accession no. GWHHMOC00000000.1). All raw data used in this paper were submitted to NGDC Bioproject database (accession no. PRJCA055833). Genome assembly and annotation, transcriptome, and metabolome data described in this article were submitted to Figshare (https://doi.org/10.6084/m9.figshare.31062166).

## References

[1] Körner C . Concepts in Alpine plant ecology. Plants. 2023;12:2666 37514280 10.3390/plants12142666 PMC10386573

[2] Xu G, Guo J, Yu X. et al. Multi-omics analysis reveals adaptive strategies of *Meconopsis horridula* to UV-B radiation in the Qinghai-Tibet plateau. Plant Cell Environ. 2025;48:8249–63 40808268 10.1111/pce.70117

[3] Lunau K, Camargo MGG, Ren Z-X. Bees, flowers and UV. Plant Biol. 2025;27:948–61 40401778 10.1111/plb.70050 PMC12477309

[4] Guo L, Winzer T, Yang X. et al. The opium poppy genome and morphinan production. Science. 2018;362:343–7 30166436 10.1126/science.aat4096

[5] Catania T, Li Y, Winzer T. et al. A functionally conserved *STORR* gene fusion in Papaver species that diverged 16.8 million years ago. Nat Commun. 2022;13:3150 35672295 10.1038/s41467-022-30856-w PMC9174169

